# Treatment of type 1A endoleak using coil embolization: a case report

**DOI:** 10.1590/1677-5449.180130

**Published:** 2019-07-03

**Authors:** Sergio Quilici Belczak, Guilherme Delicato Pedroso, Lara Cote Ogawa, Paula Thume Campos, Andre Lopes Padula, Glenna Paulain Machado, Matheus Zago Soares dos Santos, Beatriz Marques Abrão

**Affiliations:** 1 Centro Universitário São Camilo – CUSC, São Paulo, SP, Brasil.; 2 Instituto de Aprimoramento e Pesquisa em Angiorradiologia e Cirurgia Endovascular – IAPACE, São Paulo, SP, Brasil.

**Keywords:** endoleak, therapeutic embolization, treatment

## Abstract

In a type 1A endoleak, the endograft is unable to fully seal the proximal aneurysm neck and blood flow leaks between the wall of the aortic neck and the graft material. This article reports a case in which coil embolization was used and presents a literature review (PubMed, LILACS, and SciELO). Searches were run for articles published in the past 5 years using the descriptors “endoleak 1A”, “coil embolization,” and “treatment”. Type 1A endoleak occurs in 1.1% of patients within 30 days of graft placement. Treatment of an endoleak is obligatory and usually consists of sealing the proximal graft neck using stents and balloons to expand the landing zone or to increase the radial force of the graft. Some studies have suggested using embolization techniques with cyanoacrylate, fibrin glue, and Onyx, demonstrating success rates that exceed 97%. However, correction of type 1A endoleak using coil embolization has seldom been described.

## INTRODUCTION

Endovascular repair of aortic aneurysms (EVAR) has become a common alternative to open surgery because it is a less invasive procedure.^1^ However, certain complications can occur, including endoleaks. These are defined as persistent filling of the aneurysm sac after endovascular repair and they can increase the risk of rupture due to expansion of the aneurysm sac.

There are five main types of endoleak. Type 1 can be proximal (1A) or distal (1B) of the site of repair. In a type 1A leak, the endoprosthesis does not completely seal the neck of the aneurysm and there is arterial leakage between the wall of the proximal aortic neck and the graft material. In type 1B, leakage occurs between the wall of the distal aortic neck and the graft material. In a type 2 leak, the sac is filled by retrograde flow through vessels. A type 3 leak involves a tear or disconnection of the endoprosthesis. A type 4 leak is usually seen at the time of deployment of the implant in anticoagulated patients, caused by graft porosity. In type 5, the sac expands in the absence of a visible endoleak (endotension).^2^

Treatment is obligatory in cases of type 1 endoleaks,^3^ with the objective of improving the seal to the proximal implant, primarily by using stents and balloons to widen the landing zone or increase the radial force of the graft. This report describes a case in which coil embolization was used to repair a type 1A endoleak, which is a technique that has rarely been described according to a review of the literature.

## CASE DESCRIPTION

A 72-year-old patient, assessed as in regular general condition, was admitted with abdominal pains. Tomography diagnosed a 6.4 cm abdominal aortic aneurysm with a 1.1 cm proximal neck that was conical, with a 60° angle. There was also an aneurysm involving the right internal iliac, with a diameter of 3.4 cm and no distal neck. This anatomy, with the internal iliac aneurysm extending deep into the pelvis, ruled out any possibility of open surgery. During planning, the decision was taken to use an endoprosthesis long enough to reach the external iliac, since treatment with embolization was necessary because of the large aneurysm of the internal iliac with no distal neck.

The patient was therefore treated using endovascular techniques to implant an Ovation® endoprosthesis (Endologix, Irvine, California). Control angiography showed significant leakage between the endoprosthesis and the proximal aorta neck (a type 1A endoleak). The decision was then taken to use controlled-release coils to seal the area of leakage. Control angiography showed that the endoleak had been sealed once the coils were deployed.

**Figure 1:**
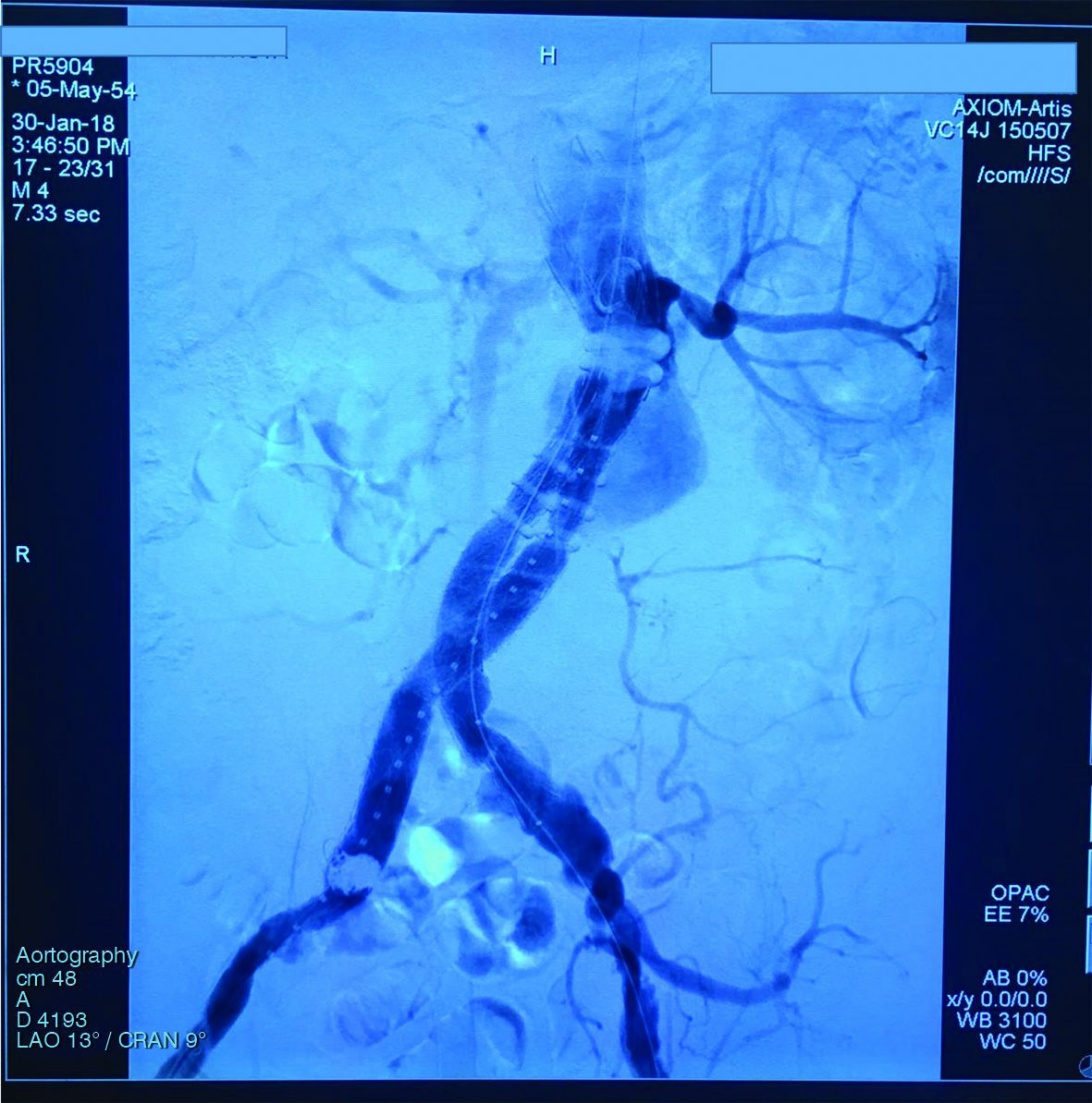
Type 1A endoleak.

**Figure 2:**
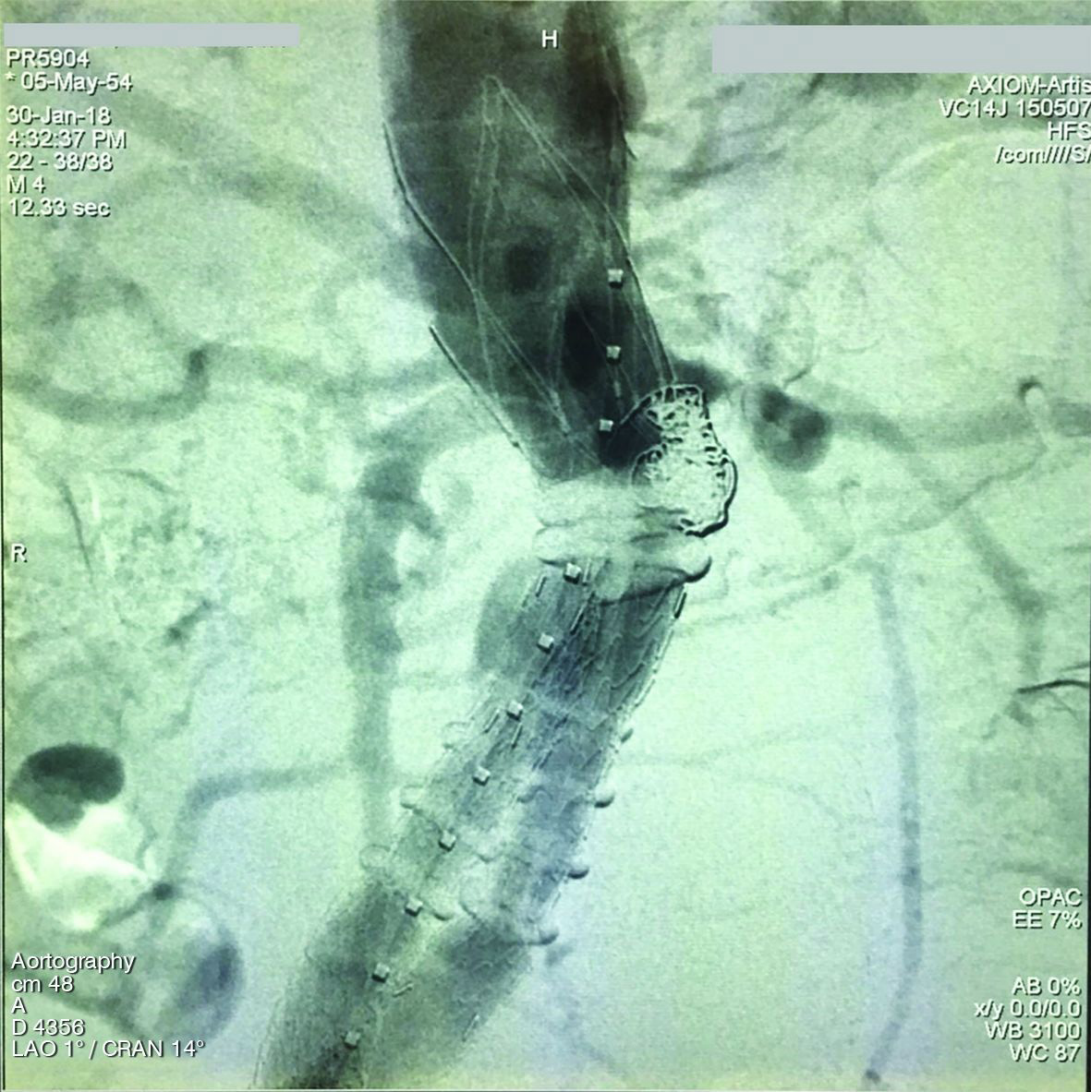
Type 1A endoleak after coil embolization.

Embolization of the endoleak was accomplished by placing a 5 Fr Simmons® catheter in contact between the endoprosthesis and the aorta and then inserting a Maestro® microcatheter through the first catheter and advancing it up to the site requiring embolization, where six Complex Trufill 3D coils were released. The patient exhibited resolution of the endoleak and was discharged from hospital after 3 days (Figures 1 and 2).

## DISCUSSION

Literature found by searching the PubMed, LILACS, and SciELO databases was reviewed. Articles published during the previous 5 years were identified using the descriptors “endoleak 1A”, “coil embolization”, and “treatment”.

The most common complication associated with endovascular treatment of aneurysms is internal leakage (endoleak), with incidence rates in the range of 6 to 57% reported in the literature. An endoleak is a failure of the implanted stent to exclude the aneurysm sac from the systemic circulation and can cause the aneurysm sac to expand and rupture. Management of endoleaks varies depending on type. Types 1 and 3 require treatment, whereas type 2 is increasingly managed expectantly, especially if there is no expansion of the aneurysm sac.^4^

The incidence of type 1 endoleaks may be attributable to the surgeon’s surgical skills, but it can also be related to skill at preoperatively sizing the endoprosthesis. This is a difficult task when the aneurysms to be repaired present complex anatomic characteristics, such as a short proximal aneurysm neck, reverse thinning of the neck, mural calcification or thrombus, and accentuated neck angles.^5^

Not all aortic aneurysms can be resolved satisfactorily. Studies have identified a direct association between short proximal aortic necks and EVAR failure, i.e., presence of endoleaks.^6^ Special techniques are therefore needed to solve these complex cases.

Although post-EVAR rupture of an aortic aneurysm involves a major risk of loss of life and demands immediate diagnosis and emergency intervention, there are no specific guidelines on the most appropriate management of this situation. Limited data from small case series indicate that these patients are most often treated with surgery. However, the risk of operating on abdominal aortic aneurysms that have ruptured post-EVAR is high and these patients generally have significant comorbidities. Endovascular repair is therefore the first-choice option if the patient’s conditions are favorable.^7^

Conversion to open surgery generally involves surgical exposure of the aneurysm, proximal and distal vascular control, complete removal of the endoprosthesis and substitution with an aortic prosthesis. Such a complex procedure is associated with significant morbidity and mortality .

Conventional methods for treatment of type 1 leakage include proximal aortic extensions, balloon angioplasty, and stents. Some cases are not suitable for endovascular reintervention and open surgical repair is the only option.^4^ EVAR with a chimney graft (Ch-EVAR) may be an option in cases with compromised cardiac function,^8^ while fenestrated stents (FEVAR) can be useful in patients with complex aortic anatomy.^9^ Successful cases of type 1A endoleak repair have been described in the literature using the Palmaz intraoperative stent.^10^ Other studies have also reported effective management of 1A endoleaks using Onyx, with or without coils, depending on the number of gutters involved.^11^

In conclusion, EVAR is increasingly the preferred method rather than open repair in cases with favorable anatomy because it is associated with less morbidity and mortality than open repair. However, there is a greater risk of reintervention because of complications, such as endoleaks. Studies show that complications have a robust relationship with short necks, graft migration, and anatomic variants. Treatments have therefore been proposed as alternatives to EVAR and new generations of endoprosthesis with improved designs for EVAR have also been developed. The resulting more satisfactory performance has reduced the need for reintervention and led to lower morbidity and mortality after aneurysm repair. Finally, despite the limited literature on the subject, studies report good short-term results. Since the techniques are recent, more time must elapse before long-term follow-up of these methods is possible.
