# Supplementary material for: Treatment of type 1A endoleak using coil embolization: a case report
Source: J Vasc Bras. 2019 Jul 3;18:e20180130. doi: 10.1590/1677-5449.180130 (PMC6636908; doi:10.1590/1677-5449.180130)
Supplement: Translation

# Translation: Tratamento de *endoleak* tipo 1A mediante embolização com molas: relato de caso

**Authors:** Sergio Quilici Belczak, Guilherme Delicato Pedroso, Lara Cote Ogawa, Paula Thume Campos, Andre Lopes Padula, Glenna Paulain Machado, Matheus Zago Soares dos Santos, Beatriz Marques Abrão

**Affiliations:** 1 Centro Universitário São Camilo – CUSC, São Paulo, SP, Brasil.; 2 Instituto de Aprimoramento e Pesquisa em Angiorradiologia e Cirurgia Endovascular – IAPACE, São Paulo, SP, Brasil.

**Keywords:** endoleak, embolização terapêutica, tratamento

## Abstract

No *endoleak* tipo 1A, a endoprótese não sela completamente o colo do aneurisma proximal, e o fluxo arterial está presente entre a parede do colo aórtico e o material do implante. Este é um relato de um caso no qual foi utilizada embolização com molas, associado a uma revisão de literatura (PubMed, LILACS e SciELO). Foram pesquisados artigos publicados nos últimos 5 anos com os descritores “endoleak 1A”, “coil embolization” e “treatment”, combinados de formas aleatórias, sendo encontrados 25 artigos. O tipo 1A ocorre em 1,1% dos pacientes após 30 dias do implante. O tratamento consiste em aumentar a vedação do implante proximal, principalmente com o uso de stents e balões para alargar a zona de aterragem ou aumentar a força radial do implante. Alguns trabalhos sugerem técnicas de embolização com cianoacrilato, cola de fibrina e uso de Onyx, mostrando taxas de sucesso superiores a 97%. Contudo, a correção de *endoleaks* tipo 1A mediante embolização com molas é pouco descrita.

## INTRODUÇÃO

O reparo endovascular de aneurisma aórtico (*endovascular aneurysm repair*, EVAR) tornou-se uma alternativa comum à cirurgia aberta, por tratar-se de um procedimento menos invasivo^1^. Entretanto, algumas complicações podem acontecer, como o *endoleak*, definido como persistência de enchimento do saco aneurismático após a correção endovascular, aumentando o risco de ruptura por expansão do saco aneurismático.

Existem cinco tipos principais de *endoleaks*. O tipo 1 é o proximal (1A) ou distal (1B) ao local do reparo. No 1A, a endoprótese não sela completamente o colo do aneurisma, e o extravasamento arterial está presente entre a parede do colo aórtico proximal e o material de implante. No 1B, o extravasamento está presente entre a parede do colo aórtico distal e o material do implante. No tipo 2, há preenchimento do saco por fluxo retrógrado nos vasos. No tipo 3, há ocorrência de rasgo ou desconexão da endoprótese. Já o tipo 4 é usualmente visto no momento da implantação em pacientes anticoagulados devido à porosidade do implante. No tipo 5, há aumento do tamanho do saco na ausência de visibilidade do *endoleak* (endotensão)^2^.

O tratamento do *endoleak* tipo 1 é obrigatório^3^ e aumenta a vedação do implante proximal, principalmente com o uso de stents e balões para alargar a zona de aterragem ou aumentar a força radial do implante. O presente trabalho relata um caso no qual foi usada embolização com molas para resolução de *endoleak* tipo 1A, técnica pouco descrita na revisão de literatura.

## DESCRIÇÃO DO CASO

Um paciente de 72 anos, avaliado com estado geral regular, foi internado com quadro de dor abdominal. Foi realizada uma tomografia, que diagnosticou um aneurisma de aorta abdominal de 6,4 cm e colo proximal de 1,1 cm, cônico, com angulação de 60°. Havia também a presença de um aneurisma de ilíaca interna direita de 3,4 cm de diâmetro sem colo distal. Essa anatomia, com o aneurisma de ilíaca interna se aprofundando na pelve, descartou a possibilidade de indicação de cirurgia aberta. Optou-se, no planejamento, por colocar endopótese com extensão permitindo alcançar a ilíaca externa, uma vez que o grande aneurisma de ilíaca interna sem colo distal impôs o tratamento mediante embolização.

O paciente foi submetido, então, ao tratamento endovascular com implante de endoprótese Ovation® (Endologix, Irvine, Califórnia). Na angiografia de controle, foi observado importante vazamento entre a endoprótese e o colo proximal na aorta (*endoleak* tipo 1A). Optou-se pelo implante de molas de liberação controlada para fechamento da área de extravasamento. Realizou-se uma angiografia de controle, que mostrou o fechamento do *endoleak* com as molas implantadas.

Para realização da embolização do *endoleak*, foi colocado um cateter Simmons® de 5 Fr encostado entre a endoprótese e a aorta. Assim, um microcateter Maestro® passou por dentro do outro cateter até alcançar o local onde se deveria embolizar, com posterior liberação de seis molas Complex Trufill 3D. O paciente apresentou resolução do *endoleak* e recebeu alta do hospital após 3 dias (Figuras 1 e 2).

**Figura 1:** *Endoleak* tipo 1A.

**Figura 2:** *Endoleak* tipo 1A após embolização com molas.

## DISCUSSÃO

Para a revisão de literatura, foram utilizadas as bases de dados PubMed, LILACS e SciELO. Foram pesquisados artigos publicados nos últimos 5 anos com os seguintes descritores: “endoleak 1A”, “coil embolization” e “treatment”.

A complicação mais comum associada ao aneurisma endovascular é o vazamento interno, com taxas de incidência entre 6% e 57% relatadas na literatura. Um *endoleak* representa falha do implante de stent para excluir o saco aneurismático da circulação sistêmica e pode levar ao aumento e ruptura do saco do aneurisma. O manuseio de um vazamento interno varia de acordo com o tipo. Os tipos 1 e 3 requerem tratamento, enquanto o tipo 2 é cada vez mais gerenciado com expectativa, especialmente se não estiver associado ao alargamento do saco aneurismático^4^.

A incidência de vazamento interno tipo 1 pode ser atribuída à habilidade cirúrgica do cirurgião, mas também à habilidade de dimensionamento pré-operatório da endoprótese. Este é dificultado quando os aneurismas a serem reparados apresentam características anatômicas complexas, como um colo de aneurisma de curto comprimento proximal, afilamento reverso do colo, calcificação mural ou trombo e acentuada angulação do colo^5^.

Nem todos os aneurismas aórticos conseguem resoluções satisfatórias. Estudos identificaram uma associação direta entre colos aórticos proximais curtos e falha do EVAR, ou seja, presença de vazamento interno^6^. Diante disso, é preciso desenvolver técnicas especiais para resolver esses casos complexos.

Embora a ruptura de aneurisma aórtico pós-EVAR represente um grande risco de vida que requer diagnóstico imediato e intervenção emergencial, não há diretrizes específicas para a gestão mais adequada dessa situação. Dados limitados de pequenas séries de caso indicam que esses pacientes são tratados com cirurgia mais frequentemente. No entanto, o risco operatório de aneurismas da aorta abdominal rompidos pós-EVAR é alto e tais pacientes geralmente apresentam comorbidades significativas. Por essa razão, o reparo endovascular é a conduta de escolha quando favorável a cada paciente^7^.

A conversão para cirurgia aberta geralmente envolve exposição cirúrgica do aneurisma, controle vascular proximal e distal, remoção completa da endoprótese e substituição com prótese aórtica. Um procedimento tão complexo acarreta morbidade e mortalidade significativas.

Métodos de tratamento convencionais para vazamento tipo 1 incluem o uso de extensões aórticas proximais, angioplastia com balão e stents. Alguns casos não são passíveis de reintervenções endovasculares, e o reparo cirúrgico aberto é a única opção^4^. EVAR com implante de chaminé (Ch-EVAR) pode ser uma opção em casos de função cardíaca comprometida^8^, bem como endopróteses fenestradas (FEVAR), que podem ser úteis em pacientes com anatomia aórtica complexa^9^. Casos de sucesso de reparo de *endoleak* tipo 1A já foram relatados na literatura e incluem uso de stent intraoperatório Palmaz^10^. Outros estudos também relataram manejo eficaz de *endoleak* 1A com uso de Onyx com ou sem molas, dependendo da quantidade de calhas envolvidas^11^.

Em conclusão, o EVAR é um método cada vez mais preferível em relação à correção aberta nos casos com anatomia favorável, por apresentar menor morbimortalidade quando comparado à correção aberta. No entanto, foi demonstrado um maior risco de reabordagem por complicações, como os *endoleaks*. Estudos mostraram forte relação das complicações com colo curto, migração da prótese e variações anatômicas. Com isso, surgiram tratamentos alternativos ao EVAR, bem como novas gerações de endoprótese com melhorias no design do EVAR. Assim, performances mais satisfatórias diminuíram a necessidade de reabordagem e garantiram menor morbimortalidade na correção dos aneurismas. Por fim, apesar da literatura limitada, os estudos evidenciaram uma boa resposta em curto prazo; por ser uma técnica recente, existe a necessidade de um maior tempo para realização de acompanhamento em longo prazo desses métodos.
